# Utilizing museomics to trace the complex history and species boundaries in an avian-study system of conservation concern

**DOI:** 10.1038/s41437-022-00499-0

**Published:** 2022-01-26

**Authors:** Mario Ernst, Knud A. Jønsson, Per G. P. Ericson, Mozes P. K. Blom, Martin Irestedt

**Affiliations:** 1 Museum für Naturkunde, Leibniz-Institut für Evolutions- und Biodiversitätsforschung, Invalidenstraße 43, DE 10115 Berlin, Germany; 2 Natural History Museum of Denmark, University of Copenhagen, Universitetsparken 15, DK 2100 Copenhagen, Denmark; 3 Department of Bioinformatics and Genetics, Swedish Museum of Natural History, Frescativägen 40, SE 104 05 Stockholm, Sweden

**Keywords:** Taxonomy, Phylogenomics, Population genetics, Speciation, Genetic hybridization

## Abstract

A taxonomic classification that accurately captures evolutionary history is essential for conservation. Genomics provides powerful tools for delimiting species and understanding their evolutionary relationships. This allows for a more accurate and detailed view on conservation status compared with other, traditionally used, methods. However, from a practical and ethical perspective, gathering sufficient samples for endangered taxa may be difficult. Here, we use museum specimens to trace the evolutionary history and species boundaries in an Asian oriole clade. The endangered silver oriole has long been recognized as a distinct species based on its unique coloration, but a recent study suggested that it might be nested within the maroon oriole-species complex. To evaluate species designation, population connectivity, and the corresponding conservation implications, we assembled a de novo genome and used whole-genome resequencing of historical specimens. Our results show that the silver orioles form a monophyletic lineage within the maroon oriole complex and that maroon and silver forms continued to interbreed after initial divergence, but do not show signs of recent gene flow. Using a genome scan, we identified genes that may form the basis for color divergence and act as reproductive barriers. Taken together, our results confirm the species status of the silver oriole and highlight that taxonomic revision of the maroon forms is urgently needed. Our study demonstrates how genomics and Natural History Collections (NHC) can be utilized to shed light on the taxonomy and evolutionary history of natural populations and how such insights can directly benefit conservation practitioners when assessing wild populations.

## Introduction

With species and populations disappearing at an unprecedented rate (Raven and Miller 2020), accurately characterizing global biodiversity is becoming increasingly urgent. Species designation and evolutionary history are crucial aspects to be considered in conservation because this knowledge helps to assess the degree of isolation between species, identify unique lineages that preserve critical genetic diversity, and optimize management plans to maximize adaptive potential. As such, the fields of taxonomy and systematics, which have been largely based on phenotypic traits and/or small sets of genetic markers, are fundamental for the protection of biodiversity. However, with a growing ability to characterize genome-wide variation, we have become increasingly aware that single gene trees do not always align with the species tree (Maddison 1997) and that genetic divergence does not always scale with phenotypic differentiation (Dussex et al. 2018). While some distant species pairs can be indiscernible in terms of morphology, populations that do exhibit phenotypic differences can lack notable genetic differentiation (Rheindt et al. 2011). Slight changes to small portions of the genome can have a large phenotypic effect, as demonstrated by the increasing number of genome-wide studies that show high levels of genetic homogeneity between species displaying striking differences in plumage coloration (Poelstra et al. 2014; Toews et al. 2016; Campagna et al. 2017). Discordance between genetic and phenotypic data can have severe implications for conservation, since a taxonomic classification that under- or overestimates genetic divergence leads to inappropriate delineation and prioritization of conservation units (Stanton et al. 2019).

High-throughput sequencing is now a well-established tool in conservation science (McMahon et al. 2014) and genomics offers powerful ways to characterize the patterns and evolutionary processes shaping lineage diversity (Supple and Shapiro 2018). With sequencing costs having decreased substantially over the past decade (Schloss et al. 2020), empiricists are no longer limited by sequencing capacity. Instead, access to a representative collection of samples, that covers geographic spread and intraspecific phenotypic variation, can be a major limiting factor and is particularly challenging for species of conservation concern (Blom 2021). Endangered species are frequently elusive and have small population sizes. This often impedes the sampling needed to study demography, biogeography, and population connectivity (Thompson 2013). Furthermore, endangered species tend to be vulnerable to human impact and, as such, the use of invasive sampling poses ethical challenges (Walters 2006; Winker et al. 2010; Costello et al. 2016). Therefore, while the technology may be in place, studying species of conservation concern remains a challenging and costly exercise that is frequently accompanied by ethical considerations.

Natural History Collections (NHCs) are important repositories of biodiversity across time and space, and represent a unique resource for the study of endangered taxa (Payne and Sorenson 2002; Wandeler et al. 2007; Bi et al. 2013; Holmes et al. 2016). Furthermore, historic specimens are testimonies of past life on earth and therefore important references to which contemporary specimens can be compared in order to understand how recent disturbance has affected wild populations. For instance, they have been successfully used to detect temporal trends in demography and genetic diversity (Miller and Waits 2003; Mondol et al. 2013). However, a major drawback of historical samples is that their DNA often suffers from postmortem DNA fragmentation and damage (Burrell et al. 2015), which can complicate the application of genomic methods. To analyze whole-genome datasets, researchers strongly rely on existing genomic resources such as high-quality genome assemblies to which degraded DNA can be compared. Even though such resources have remained largely absent for many taxa, recent developments make it increasingly tractable to generate reference genomes de novo, which permits the wide-scale utilization of NHCs in the study of endangered taxa. Here, we use linked-read sequencing to assemble a de novo reference genome and use whole-genome resequencing of historical specimens to study the evolutionary history and taxonomic status of an avian species complex from Asia.

**Fig. 1:**
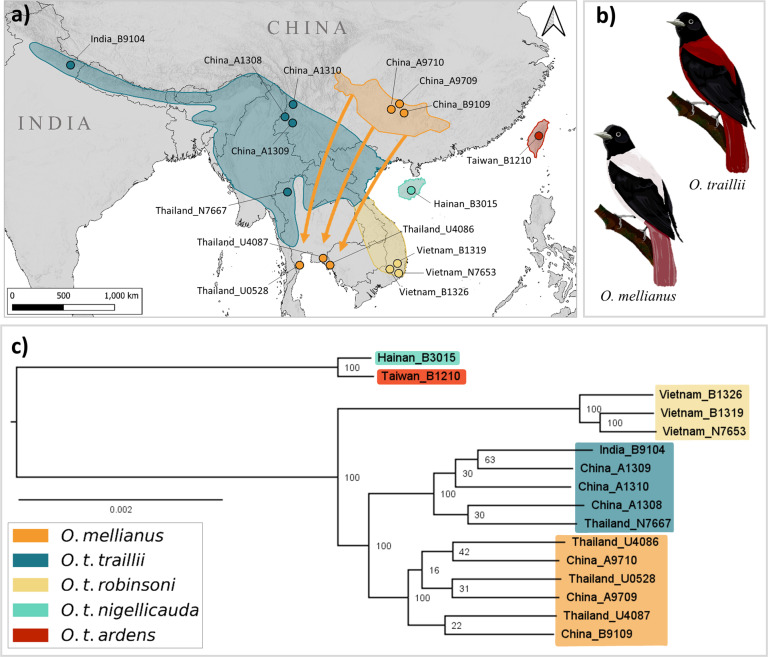
Sampling localities, plumage variation and nuclear phylogeny. **a** Map showing the breeding ranges of *O. mellianus* and the *O. traillii* subspecies (*O. t. traillii, O. t. robinsoni, O. t. nigellicauda*, and *O. t. ardens*) according to BirdLife International (2016, 2018). Colored dots represent sampling locations and are tagged with their corresponding sample ID. The arrows depict estimated migration routes of *O. mellianus*. **b** Illustrations representing variations in plumages of *O. traillii* (upper) and *O. mellianus* (lower). **c** Best-scoring maximum-likelihood tree obtained from the concatenated nuclear sequences. The scale bar is measured in absolute divergence-time units. Taxa are colored according to the colors assigned in the distribution map.

The endangered silver oriole, *Oriolus mellianus*, is a migratory songbird of the family Oriolidae and known to breed only in a few localities in South-Central China. Its wintering grounds are poorly known, with some records from Thailand and a few records from South-West Cambodia. Loss and fragmentation of forest habitat throughout the species’ range is likely responsible for the declining trajectory of this species, which today has an estimated population size between 1000 and 2499 individuals (BirdLife International 2016). Based on morphology, *O. mellianus* has been considered sister to the polytypic maroon oriole, *O. traillii*, with an extensive distribution across South-East Asia (Fig. 1a). Male individuals display striking phenotypic differences, as *O. mellianus* specimens are coated silvery white in body parts where *O. traillii* has different hues of crimson maroon (Fig. 1b). Despite these plumage differences, recent phylogenetic analyses based on a few genetic markers have suggested that *O. mellianus* may be nested within *O. traillii* and perhaps even within the geographically adjacent subspecies *O. t. traillii* (Jønsson et al. 2019). However, the study by Jønsson et al. (2019) included a single *O. mellianus* individual, making it challenging to discriminate between competing hypotheses that could explain the observed branching patterns, such as recent phenotypic divergence or hybridization between distinct morphological forms.

Population-wide sampling and whole-genome sequencing provides a more detailed view on the evolutionary history and taxonomy of this species group. As these genomic resources were previously nonexisting, we generated the first reference assembly and whole-genome population-level dataset for members of the Oriolidae family. This allowed us to assess if *O. mellianus* represents a phylogenetically valid species and establish the systematic affinities of *O. mellianus* relative to other populations of *O. traillii*. Finally, we quantified the extent of ancient and extant population connectivity and searched for genomic regions of differentiation to locate candidate genes of importance for the development of the distinct plumage characters of *O. mellianus*. These results have direct implications for the conservation management and status of Asian orioles, underline how modern genomic approaches can provide a genome-wide view on population connectivity, and how NHCs represent a powerful resource for the study of endangered taxa.

## Materials and methods

### Sample collection, DNA extraction, library preparation and sequencing

No high-quality genome assembly was available for any oriole species. Thus, we assembled a de novo genome to use as a reference for the whole-genome resequencing data sourced from museum specimens. For the *de novo* genome assembly, we extracted DNA from a blood sample taken from a closely related black-hooded oriole (*Oriolus xanthornus* subsp. *maderasperatus*) individual sampled in India (ZMUC139617). DNA was extracted using the KingFisher DNA extraction platform. Subsequently, a linked-read sequencing library was prepared using the Chromium Genome platform (10X Genomics) and sequenced on the HiSeqX at SciLifeLab Uppsala (Sweden).

Furthermore, we generated whole-genome resequencing data for sixteen museum specimens collected between 1906 and 1952: six *O. mellianus* and ten *O. traillii* individuals, representing all currently recognized maroon oriole subspecies (five *O. t. traillii*, three *O. t. robinsoni* samples, one *O. t. nigellicauda*, and one *O. t. ardens*; Table S1). Based on geographic location and collection availability, we selected samples collected across South-East Asia (Fig. 1a). We sourced genetic material from toepads of museum-study skins, extracted DNA following the protocol described in Irestedt et al. (2006), and prepared four Illumina libraries per individual following the protocol published by Meyer and Kircher (2010). For each sample, we pooled the libraries in equimolar ratios before sending them to SciLifeLab Stockholm (Sweden) for paired-end sequencing (read length: 150 bp) using Illumina Next Generation Sequencing technology on the HiSeqX or NovaSeq sequencing platform.

### De novo 10X chromium assembly

For the assembly of the *O. xanthornus* draft genome, the NextFlow-core pipeline Neutronstar was used. This is a best-practice assembly pipeline for the de novo assembly and quality control of 10x Genomics Chromium data. 10X Genomics linked-reads were assembled using Supernova, and in order to assess assembly quality and completeness, standard quality metrics were computed using QUAST (V5.0.2; Gurevich et al. 2013). In addition, the presence, completeness, and copy number of Benchmarking Universal Single-Copy Orthologs (BUSCOs) was evaluated using BUSCO (V3.0.2; Simão et al. 2015). For evaluation, the output of these assessment tools was visualized in a MultiQC (v1.7; Ewels et al. 2016) report.

### Cleaning

Following sequencing, we polished the raw resequencing data using a custom-designed workflow for museum specimens (https://github.com/MozesBlom/NGSdata_tools/). This workflow consists of (i) removal of duplicates using Super-Deduper (V1.0.0; Petersen et al. 2015), (ii) adapter trimming using Trimmomatic (V0.36; Bolger et al. 2014), (iii) merging of overlapping read pairs with PEAR (V0.9.10; Zhang et al. 2014a), (iv) quality trimming with Trimmomatic (V0.36), and (v) removal of low-complexity reads using an in-house-developed Python script. Before and after the cleaning, we visually inspected the quality of the reads using FastQC (V0.11.5; Andrews 2010).

### Mitogenome reconstruction

By blasting the whole mitogenome of *O. chinensis* (accession number: JQ083495) against the *de novo* genome assembly of *O. xanthornus* with Blastn (Zhang et al. 2000), we identified a single contig (ID 118416) scoring the complete mitogenome. After visually inspecting and curating this full *O. xanthornus* mitogenome sequence using Geneious (V2019.1.3; Kearse et al. 2012), we used it as a reference for reconstructing the mitogenomes of all focal individuals. Mitogenomes were reconstructed from the cleaned read data using MITObim (V1.9; Hahn et al. 2013), which uses an iterative baiting and mapping strategy and has been shown to effectively reconstruct mitogenomes even when there is no reference sequence for the target species (Hahn et al. 2013). After reconstructing the mitogenomes, the control region was excluded because of its complex evolutionary properties that hamper its reconstruction (Howell et al. 2007).

### Mitochondrial phylogeny

Because of the linked nature of the mitogenome, we treated it as a single hereditary unit for phylogenetic inference. We selected the best-scoring tree out of 10 maximum-likelihood tree searches in RAxML (V8.2.10; Stamatakis 2014) and calculated the bootstrap support for each node with 1000 bootstrap replicates. For the phylogenetic inference, we selected the general time reversible (GTR) model of evolution with a gamma-distributed rate variation among sites based on the Bayesian information criterion provided by MEGA X (Kumar et al. 2018).

### Mapping

In order to map the cleaned reads to the *O. xanthornus* reference genome, we used the BWA-MEM algorithm (V0.7.17; Li 2013). Then, we used SAMtools (V0.1.19; Li et al. 2009) to (i) convert the SAM files into BAM format, (ii) remove reads that did not pair properly with the reference (iii), sort and index the BAM files, and (iv) merge the library-specific BAM files by individual. Since short scaffolds have a higher likelihood to include assembly errors and are frequently repetitive in nature, we filtered each BAM file to exclusively keep reads mapping to the 1000 longest scaffolds (representing ~85% of the total genome size) for downstream analyses. The shortest scaffold included for downstream analyses spanned 41,402 bp. Last, we evaluated DNA damage in the final BAM files by using mapDamage (V2.0; Jónsson et al. 2013).

### Variant calling and filtering

We performed variant calling and filtering using two alternative workflows based either on “hard-calling” genotypes or on inferring genotype likelihoods (GLs). The latter are able to incorporate statistical uncertainty through downstream analyses and recommended when working with low-coverage data, as is typically the case with historical samples (Korneliussen et al. 2014). For the GL approach, we used ANGSD (V0.921; Korneliussen et al. 2014) to jointly call biallelic SNPs. GLs were estimated using the GATK model (McKenna et al. 2010). For that calculation, we considered only bases with a quality score of at least 20 from reads that have a minimum mapping score of 20 and mapped to unique positions. Furthermore, we set the SNP *p*-value threshold to 1e–6, which has been reported as a conservative value (Korneliussen et al. 2014) and rejected sites with a significant *p*-value for being triallelic. Similarly, we discarded sites that had an individual coverage below 10 or above 100 reads, missing data for two or more individuals or a minor allele frequency (MAF) below 0.12. While the latter may lead to the removal of subspecies-specific alleles in *O. t. ardens* and *O. t. nigellicauda*, we decided for a stringent MAF threshold because we are dealing with medium-coverage data that show degradation patterns (Table S1; see below). In order to perform downstream analyses (NGSadmix and PCAngsd) on different sample subsets, we ran ANGSD twice: once including the total sample pool and once including only *O. mellianus*, *O. t. robinsoni*, and *O. t. traillii*, which we henceforth refer to as continental taxa.

For the hard-calling pipeline, we employed FreeBayes (V1.0.2; Garrison and Marth 2012) using the joint genotyping model with a population prior. Similar to the previous approach, genotypes were called exclusively considering bases with a phred quality score above 20 from reads with a mapping quality score above 20. Subsequently, we applied analogous filtering steps to the previous approach using VCFtools (V0.1.15; Danecek et al. 2011) and Vcflib (Garrison 2012). More specifically, we filtered variants by (i) eliminating individual genotypes if the depth was less than 10 or more than 100 reads per individual, (ii) removing sites if more than a single individual had missing data, (iii) decomposing complex variants into SNPs and indels before removing all variants that were not SNPs, (iii) eliminating nonbiallelic sites (including the reference allele), (iv) removing variants with more than one missing individual, and (v) dismissing variants with a MAF below 0.12 so that at least 4 chromosomes had to support the minor allele.

### Phylogenetic framework and divergence-time estimation

We employed two distinct approaches to infer a nuclear phylogeny: a maximum-likelihood approach based on a genome-wide concatenated dataset and a full-coalescent approach based on a subset of unlinked biallelic SNPs. In order to infer the nuclear concatenated tree, we first generated consensus sequences for each individual based on the filtered VCF files by using BCFtools (V1.3.1; Li and Durbin 2009). Heterozygous sites were included and coded according to the IUPAC-ambiguity code. However, we masked sites if the coverage was below 10 reads or above three times the mean coverage of the individual under consideration. This was done by computing read depth at each position using SAMtools (V0.1.19) and subsequently masking the individual consensus sequences using BEDtools (V2.27.1; Quinlan and Hall 2010). Next, we extracted 10 kb windows in a 90 kb distance if the contig length exceeded 100 kb. For each window to be included, at least 50% of the positions had to have sequence data for at least 50% of all individuals. Ultimately, we based the phylogenetic inference on a concatenation of 9738 (10 kb) windows that passed these requirements. Then, we inferred a phylogeny by performing 20 maximum-likelihood tree searches using 10 random and 10 parsimony-based starting trees. These inferences were done under the GTR model of evolution for unphased diploid genotype data and default settings. In addition, we performed nonparametric bootstrap by resampling alignment columns and reinferring the tree for 100 replicates. We then computed branch-support values onto the best-scoring tree. Furthermore, we used Twisst (Martin and Van Belleghem 2017) to quantify the frequency of competing subtree topologies and estimate the number of windows supporting different phylogenies. The input file contained gene trees constructed based on the windows selected for the concatenated tree inference. For each window alignment, we performed a maximum-likelihood tree search using RAxML-NG (Kozlov et al. 2019) under the GTR model of evolution for unphased diploid genotype data. Last, in order to estimate divergence times, we calculated uncorrected sequence divergences (*p*-distances) between the samples from the concatenated dataset using DiStats (Astrin et al. 2016). Subsequently, we scaled the sequence-divergence estimates with an empirically informed mutation rate for birds (Zhang et al. 2014b; Smeds et al. 2016) to approximate the pairwise divergence times between all individuals (number of generations, Table S2).

Since concatenated analyses do not account for incomplete lineage sorting (ILS), we also used SNAPP (Bryant et al. 2012) to infer the species-tree under a full-coalescent model. This approach assumes that the loci are biallelic and unlinked. Whereas the former requirement is already met due to our filtering criteria, we thinned the variants by setting the minimum separation criterion between SNP positions to 10 kb in order to minimize linkage disequilibrium (Christmas et al. 2021). Next, we converted de VCF file into a binary nexus matrix using a custom python script (Ortiz 2019). The final SNAPP input file contained 84,236 SNPs. We used Beauti to generate a BEAST XML file in which we (i) assigned each individual to its respective population based on the results obtained by NGSadmix and PCAngsd (see below), (ii) fixed coalescence rate at 10 and forward and reverse mutation-rate parameters to 1, (iii) set the chain length for the MCMC to 1*10-e7 generations, and (iv) sampled trees every 1000th iteration. After the run, we discarded the first 10% of the chain as burn-in using TRACER (V1.7.1; Rambaut et al. 2018) and we assumed convergence of parameter estimates if the respective effective sample size was greater than 200.

### Testing for hybridization

We took three alternative approaches to evaluate whether the observed incongruence between phenotypic and genetic similarity was caused by (a) recent speciation and phenotypic divergence or (b) hybridization between distinct morphological forms. First, we used PCAngsd (V0.98; Meisner and Albrechtsen 2018) to run a principal component analysis (PCA) and characterize the genetic structure among subspecies and species. The data were plotted following a custom python script (https://plot.ly/python/v3/ipython-notebooks/principal-component-analysis/). Second, we used a clustering identification approach to estimate admixture proportions across individuals. We ran NGSadmix (Skotte et al. 2013) 15 times for K-values ranging from 1 to 10 and with the remaining settings left at their default. The optimal K-value was determined based on the DeltaK statistic (Evanno et al. 2005), which we calculated from the obtained log-likelihood scores using CLUMPAK (Kopelman et al. 2015). In order to ensure that any potential substructure within continental taxa is detected, we ran independent population-structure analyses once for all individuals and once including only *O. mellianus*, *O. t. traillii*, and *O. t. robinsoni*. Finally, unbalanced allele-sharing in a four-taxon set [((P1,P2),P3),O] can be a sign of post-speciation gene flow (ABBA–BABA test/Patterson’s D; Durand et al. 2011). Building further on the inferred species-tree and cluster identification, we calculated Patterson’s D using doAbbababa2 (Soraggi et al. 2017) to evaluate several putative hybridization scenarios within continental taxa. While *O. t. nigellicauda* is a visitor to Vietnam (Mason and Allsop 2009), *O. t. ardens* is not known to migrate to continental territory and geographically more isolated. Therefore, we chose *O. t. ardens* as outgroup (O) in all comparisons. Then, we computed D-statistics for all possible individual-level comparisons (including one individual per group) and the only possible population-level comparison (including multiple individuals per group). For the individual-based tests, P1 and P2 always consisted of samples belonging to the same (sub-)species, and P3 consisted of a sample belonging to a different taxon as shown in Fig. S1. For the population-level comparison, we used the same software to test for gene-flow between continental taxa. In other words, we checked whether the whole population of *O. t. robinsoni* (P3), shares more similarities with *O. mellianus* (P1) than it does with the *O. t. traillii* (P2) population as shown in Fig. S2.

### Genome-wide differentiation between closely related distinct color morphs

**Fig. 2:**
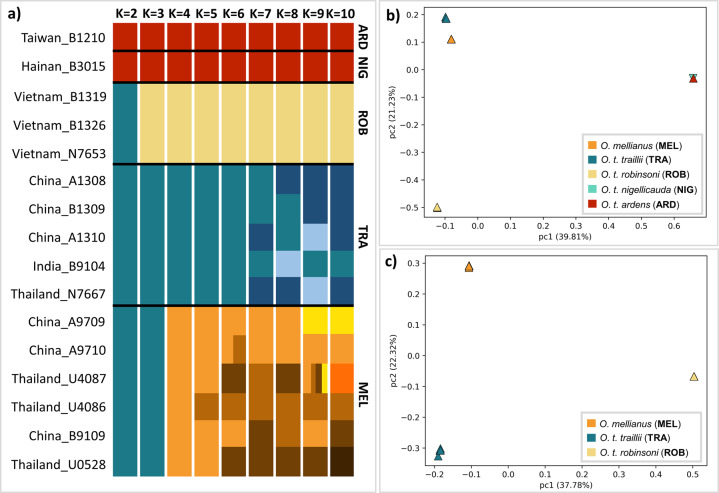
Population structure. **a** Admixture panels showing the best-scoring cluster configurations out of 15 runs produced by NGSadmix for K-values ranging from two to 10. The X axis represents the admixture proportion and the Y axis indicates the sample ID and taxon correspondance. Thick black horizontal lines draw the taxon boundaries according to current classification. Taxa are labeled according to the following abbreviations: ARD: *O. t. ardens*, NIG: *O. t. nigellicauda*, ROB: *O. t. robinsoni*, TRA: *O. t. traillii*, MEL: *O. mellianus*. Optimal number of discrete groups returned by DeltaK is 4. B and C: PCA plots showing how samples cluster along continuous axes of variation when including all samples (**b**) and after excluding island taxa (*O. t. ardens* and *O. t. nigellicauda*) (**c**).

All of the analyses indicated that *O. traillii* is a paraphyletic species group and that *O. t. traillii* is likely more closely related to the distinct morph, *O. mellianus*, than to all other *O. traillii* populations (Figs. 1c, 2, S3-6). Notwithstanding close phylogenetic association, *O. t. traillii* and *O. mellianus* strikingly differ in plumage coloration (Fig. 1b) and we therefore characterized the genetic landscape of differentiation (F_st_) to identify candidate loci underlying plumage variation. We measured differentiation by calculating mean F_st_ (Weir and Cockerham 1984) across nonoverlapping 50 kb windows using VCFtools (V0.1.15) and then visualized the obtained statistics by generating a Manhattan plot using the qqman package (V0.1.4; Turner 2014) in R (V 3.5.0; R Core Team 2018; Fig. 4). Then, we arbitrarily defined outliers as windows with Z(F_st_) values over 1.15 (the top 5%) and annotated these by blasting the window sequences to the chicken genome (Gallus.gallus.5.0.cds) using BLAST + (v2.6.0; Camacho et al. 2009). The genes within the top 5% selected windows were then compared with genes involved in plumage coloration in birds. These represent candidate gene regions under selection and thus of potential importance for the development of the distinct differences in plumage between *O. t. traillii* and *O. mellianus*.

## Results

### Data processing

The resulting 10X chromium assembly included 37,670 contigs, had a scaffold N50 of 8.3Mbp, and a pseudohaploid assembly length of 1102Mbp. This value is slightly below the genome size of the closely related species *O. chinensis*, which spans 1398Mbp (C-value = 1.43 pg; Krishan et al. 2005). Out of 303 BUSCOS, 185 (61.1%) were complete and single copy, 2 (0.7%) complete and duplicated, and 116 (38.2%) fragmented or missing. On average, each sample had 267 million raw reads, but this number dropped to 133 million after cleaning, mapping, and excluding short scaffolds (Table S1). The decrease in read numbers was largely caused by the merging of overlapping read pairs. This is expected, given the short DNA templates obtained from historical specimens and does not necessarily reflect loss of sequence information. Furthermore, we found excess of cytosine-to-thymine (C-to-T) misincorporations at 5′-termini and an excess of guanine-to-adenine (G-to-A) misincorporations at 3′-termini (Table S1). After variant calling and filtering, we retained 7.16 million SNPs with GLs and 4.12 million hard-called genotypes.

### Phylogenetic framework and divergence-time estimation

Our phylogenetic results unanimously show that *O. mellianus* forms a well-supported monophyletic lineage that is nested within *O. traillii* and that *O. t. ardens* and *O. t. nigellicauda* (which we will refer to as island subspecies in this paper) form a sister clade that is distantly related to the continental taxa (Figs. 1c, S3–4). Furthermore, the concatenated and full-coalescent phylogeny, as well as the population-structure analyses suggest that *O. mellianus* is most closely related to *O. t. traillii* (Figs. 1c, 2, S3). Moreover, this sister relationship is also supported by the mitogenome tree, even though this phylogeny renders *O. t. traillii* paraphyletic with largely unresolved relationships (Fig. S4). In slight contrast to these results, which collectively suggest that *O. mellianus* is most closely related to *O. t. traillii*, the Twisst analyses reveal substantial heterogeneity among nuclear gene trees with three topologies being almost equally frequent (Fig. S7). While *O. mellianus* is still placed within *O. traillii*, the relationship between *O. mellianus, O. t. traillii*, and *O. t. robinsoni* is therefore less certain.

Based on the nuclear concatenated dataset and the 4.6*E-9 mutation rate of flycatchers by Smeds et al. (2016), the average number of generations since the split between *O. mellianus* and *O. t. traillii* was estimated to around 127,000 generations ago (Table S2). Furthermore, the split between these two taxa and *O. t. robinsoni* was estimated to 224,000 generations ago. Notably, the divergence between *O. mellianus* and *O. t. traillii* is less than 2.5 times the estimated number of generations between the most divergent individuals within *O. mellianus* (ca 52,000 generations) and *O. t. traillii* (ca 59,000 generations), respectively. As the generation time for *O. mellianus* and *O. traillii* is unknown, we cannot estimate the absolute divergence time between major lineages within *O. traillii*. However, BirdLife International has estimated the generation length for all orioles to 3.7 years, which suggests that the split between *O. mellianus* and *O. t. traillii* is younger than 500,000 years.

### Introgression analyses

When including all individuals, structure analyses support an optimal number of four clusters, with *O. mellianus, O. t. traillii*, and *O. t. robinsoni* forming three and island subspecies grouped into a single cluster (Figs. 2a, b, S6). Similarly, when excluding island taxa, the population-structure analyses support an optimal number of three clusters (Figs. 2c, S5). In both cases, the cluster assignment displayed by the NGSadmix plots is congruent with the PCAngsd analyses (Fig. 2). Furthermore, the order in which the clusters split as the K-value increases and the relative distance between clusters in the PCA plot mirrors the phylogenetic relationships recovered by the nuclear phylogeny (Figs. 1c, 2). For example, *O. t. ardens* and *O. t. nigellicauda*, which were found to be most divergent, split off first in the admixture panels and are most distant in the PCA plot (Figs. 1c, 2a, b). *O. t. traillii* and *O. mellianus*, which were found to be more closely related, only separate at higher K-values and are adjacent in the PCA plot (Figs. 1c, 2a, b). Across all runs, NGSadmix clearly assigned each individual to its respective taxon and the PCA plot shows very tight clustering of individuals by taxon (Fig. 2b, c, S5-6), indicating that none of the individuals included in this study are recent hybrids.

**Fig. 3:**
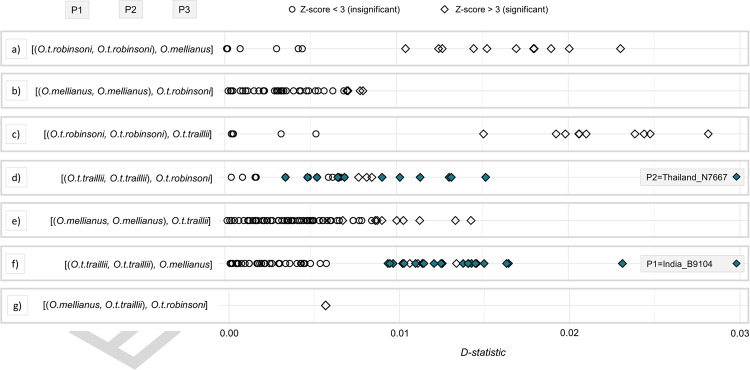
Plots showing the ABBA–BABA test results. The higher the D-statistic, the higher the excess of shared derived alleles between P2 and P3. **a**–**f** show the results from the individual-based test. Here, samples were tested individually and shuffled to compute all possible comparisons. **g** shows the obtained D-statistics from a single population-based comparison in which individuals were grouped by taxon and compared collectively. The significance of the tests is visualized using differently shaped dots. Round dots indicate that the comparison was insignificant (Z-score < 3), while squares indicate that the comparison was significant (Z-score > 3). Individuals discussed in the main text are highlighted according to the taxon-specific color-coding employed throughout the paper. Note that in **d**, we highlighted the comparisons where Thailand_N7667 was set as P2. On the other hand, in **f**, we highlighted the comparisons where India_B9104 was set as P1. The outgroup (O) was formed by *O. t. ardens* but was omitted in this figure for simplicity.

Positive D-statistics indicate introgression between P2 and P3, while negative D-statistics hint at introgression between P1 and P3. Although the D-statistics were significant for the population, as well as the individual-level comparisons, they tend to be relatively low (<0.03, Fig. 3). The results from the population-based test showed that *O. t. traillii* and *O. t. robinsoni* share significantly more site patterns than *O. mellianus* and *O. t. robinsoni* (D = 0.0056; Z-score = 3.39; Fig. 3g, Table S3). Furthermore, individual-based tests showed that specimens from the same taxon present different levels of shared derived alleles with individuals from other taxa (Fig. 3a–f, Table S3). D-statistics differed substantially, depending on individuals used (within the same species), suggesting that local populations may have differed in the timing and/or extent of hybridization. For example, when comparing *O. t. traillii* individuals to each other in order to assess if some are more or less similar to *O. t. robinsoni* (Fig. 3d), the D-statistics ranged from 0 to 0.015 and *O. t. traillii* individual Thailand_N7667 tended to be more similar. Moreover, when comparing *O. t. traillii* individuals to each other in order to contrast levels of similarity with *O. mellianus* (Fig. 3f), the D-statistics ranged from 0 to 0.023 and *O. t. traillii* individual India_B9104 tended to be less similar. While the significant excess of shared derived alleles suggests that interspecific gene flow has taken place after initial divergence, relatively low levels of genealogical discordance, clear genetic distinctiveness, and lack of contemporary admixture indicate that gene flow has ceased among contemporary taxa.

### Candidate loci for plumage coloration

**Fig. 4:**
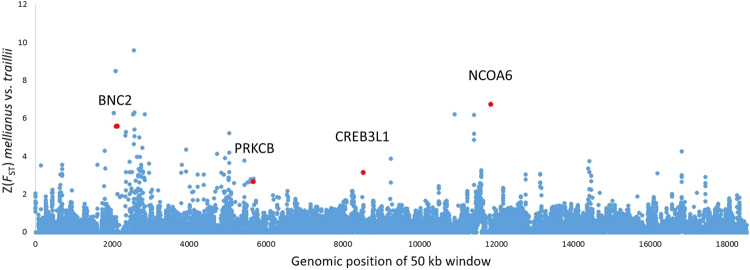
Manhattan plot showing the Weir and Cockerham’s Fst values in 50-kb windows across the whole genome between *O. t. traillii* and *O. mellianus*. Top 5% outliers that may be of importance for the development of the distinct plumage of *O. mellianus* are shown in red.

The Fst outlier analysis yielded a total of 402 candidate genes when using a 95% cutoff (Z(Fst)>1.15). Among the top 5% outliers were RPE65, ADCY7, CTCF, FCD9, WNT7A, CREB3L1, PRKCB, BNC2, and NCOA6 (Fig. 4). RPE65 is a paralog of BCO2, which is involved in carotenoid degradation to colorless derivatives in white-skinned birds (Toews et al. 2016). CREB3L1, PRKCB, BNC2, and NCOA6 are linked to pigmentation. ADCY7, CTCF, WNT7A, CREB3L1, FCD9, and PRKCB were all differentially expressed between sexes in four plumage patches in zebra finches (Sly 2019). Perhaps most relevant for *O. mellianus* is CREB3L1, which showed significant signs of differential expression in orange and gray patches in zebra finches. BNC2 and NCOA6 respectively influence skin pigmentation (Jacobs et al. 2013) and hair coloration in humans (Bonilla et al. 2020).

## Discussion

Accurately identifying evolutionary units is crucial for conservation and NHC offers a unique opportunity to study endangered taxa. In this study, we assembled a de novo genome, used it as an anchor to guide whole-genome resequencing of museum specimens, and re-evaluated the evolutionary and taxonomic status of *O. mellianus* and allied oriole species. Our results support that the endangered *O. mellianus* represents a distinct, recently emerged, and likely reproductively isolated lineage, which merits treatment as a species (De Queiroz 1999). Simultaneously, our results highlight the need for a taxonomic revision of *O. traillii* ssp., which appears to be a complex comprising several distinct species. We find strong support for a scenario in which *O. mellianus* has recently emerged from a subspecies of *O. traillii* and rapidly diversified in plumage coloration. Due to their close affinity, we were able to identify several candidate loci that underlie plumage differentiation and therefore establish a promising research system for the study of speciation.

### The systematic affinity of O. mellianus and masked diversity within O. traillii

In line with the results by Jønsson et al. (2019), our analyses show that *O. mellianus* is nested within *O. traillii* and consequently *O. traillii* represents a paraphyletic species complex. Our results consistently recovered the distinct island populations as sister taxon to the continental clade and indicate that *O. mellianus* is most likely sister to *O. t. traillii*. While the concatenation and the species-tree approach strongly support a sister relationship between *O. mellianus* and *O. t. traillii* (Figs. 1c, S3), the underlying distribution of gene-tree frequencies suggests that a more detailed evaluation is warranted. Topology weighting in the Twisst analysis found high levels of gene-tree discordance with almost equal support for three competing topologies (Fig. S7). The concatenated and Twisst analyses were based on the same dataset, but it is now well established that concatenation masks the underlying heterogeneity in coalescent patterns (Mendes and Hahn 2018). Interestingly, in contrast to concatenation approaches, SNAPP accounts for ILS but also strongly supported an *O. mellianus* and *O. t. traillii* sister relationship (Fig. S3). We therefore hypothesize that *O. mellianus* and *O. t. traillii* are indeed sisters but diverged shortly after their common ancestor split from *O. t. robinsoni*. Rapid diversification could lead to a high incidence of ILS and consequently, extensive gene-tree heterogeneity. The discordance among gene-trees was possibly also reinforced by post-divergence gene-flow (see below).

In comparison with previous research (Jønsson et al. 2019), our study includes a large number of *O. mellianus* and *O. traillii* representatives, which allows a detailed view on evolutionary relationships at an intraspecific level. By increasing the number of individuals per taxon, we were able to determine that the currently recognized species *O. mellianus* and the different subspecies of *O. traillii* (when represented by more than one specimen) form highly supported monophyletic groups according to the nuclear data (*O. mellianus* and *O. t. robinsoni* are also monophyletic according to the mitochondrial data; Fig. S4). This is concordant with our PCA and NGSadmix results that recover each nuclear monophyletic lineage as a separate cluster (Fig. 2). Our sampling for *O. t. ardens* and *O. t. nigellicauda* is relatively low and this heterogeneity in sample size can introduce biases when filtering variants (Puechmaille 2016). However, repeating population-structure analyses with a different MAF cutoff (0.05) did not change the result, suggesting that island taxa indeed form a monophyletic clade. The high bootstrap support of each clade in the nuclear concatenated tree (Fig. 1c) and the assignment of each individual to its respective cluster (Fig. 2) indicate that *O. mellianus, O. t. traillii*, and *O. t. robinsoni* accumulated a substantial amount of unique substitutions. To summarize, our exhaustive dataset confirms that these taxa are genetically cohesive and well-differentiated lineages, suggesting that they evolved independently for a considerable period of time.

Although the mitochondrial phylogeny largely mirrors the branching patterns recovered by analyses based on nuclear data (Figs. 1c, S3–4), we found instances of mitonuclear discordance. The mitogenomic data place *O. mellianus* as nested within, rather than sister to *O. t. traillii* and one *O. t. traillii* specimen (India_B9104) groups with the *O. t. robinsoni* clade (Fig. S4). This paraphyly could potentially be attributable to ILS or mitochondrial introgression, and differentiating between these two scenarios remains a challenging endeavor (Holder et al. 2001; Buckley et al. 2006 but see Andersen et al. 2020). If ILS is responsible for the observed discordance, it would require retention of shared mitochondrial haplotypes between *O. t. traillii* and *O. t. robinsoni*. Twisst points at a relatively rapid radiation of continental taxa. Such radiations are particularly prone to present high levels of ILS. Furthermore, ILS is most likely observed in taxa with large effective population sizes (Maddison 1997; Degnan and Rosenberg 2009). In this respect, it is noteworthy that *O. t. traillii* has the broadest distribution of all taxa in this study and most certainly the largest population size (Del Hoyo et al. 2008). Mitochondrial introgression, on the other hand, can lead to mitonuclear discordance by horizontally transferring the mitogenome from one taxon into the nuclear background of another. The Indian sample (India_B9104) could hypothetically be carrying an introgressed mitogenome that was donated by *O. t. robinsoni* after *O. t. traillii* and *O. t. robinsoni* diverged. Interestingly, India_B9104 shares the least number of derived alleles with *O. mellianus* and *O. t. robinsoni* compared with its conspecifics (Fig. 3f, Table S3). This observation contradicts the hypothesis of mitochondrial introgression, though mitochondrial introgression has previously been reported with negligible signs of nuclear introgression (Good et al. 2015; Andersen et al. 2020). Taken together, the mitonuclear discordance can be explained by both post-divergence gene flow and ILS. While we have detected biased excess of discordant genealogies stemming from post-divergence gene flow, we cannot discard that ILS is also leading to random gene-tree discordance. ILS and introgression are non-mutually exclusive evolutionary processes and can act together, leading to discordant coalescent histories.

### Evidence for gene-flow

Building further on our population-level sampling for several (sub-) species, we find no evidence for contemporary gene flow among lineages. However, explicit tests for introgression reported significant D-statistics (Z-score > 3; Fig. 3, Table S3). While the observed D-statistics are relatively low (D < 0.03), the ABBA–BABA analyses are parsimonious in terms of geography. For instance, the population-level comparison showed that *O. t. traillii* and *O. t. robinsoni*, which have adjacent breeding-distribution ranges, share a significant excess of derived alleles compared with *O. mellianu*s (Figs. 1a, 3g, Table S3). However, this result should be interpreted with caution, given that in this comparison, we have followed the concatenated nuclear phylogeny: [(*O. mellianus*, *O. t. traillii*),*O.t.robinsoni*]. The results of our Twisst analysis equally support the three possible in-group topologies, suggesting that this assumption may be violated (Fig. S7). Even so, individual-based tests also show geographically parsimonious signals. In these comparisons, we assessed if individuals of the same taxon share more or less similarities with another taxon. They are therefore valid under all topologies supported by Twisst. For example, the *O. t. traillii* sample India_B9104, is geographically most isolated and also has the lowest signs of introgression with *O. t. robinsoni* and *O. mellianus* (Figs. 1a, 3f, Table S3). Conversely, Thailand_N7667 is the *O. t. traillii* sample in closest geographic proximity to *O. t. robinsoni* and displays the highest signs of introgression with this taxon (Figs. 1a, 3d, Table S3).

The significance and geographic correlation of our results suggest that the observed excess of discordant genealogies may stem from a rapid radiation, followed by post-divergence gene flow between *O. t. traillii* and *O. t. robinsoni*. Ancestral hybridization, rather than recent gene flow, could explain why NGSadmix detected no admixture (Fig. 2a). The ABBA–BABA test explicitly focuses on the frequency of non-concordant site patterns and is capable of detecting historical gene flow, while NGSadmix is only sensitive to recent admixture (Skotte 2015). It could be argued that recent gene flow was not detected because of the lack of samples coming from putative hybrid zones, where admixture is more likely. Thus, we recommend that future studies include additional samples, particularly from areas where these taxa meet. However, genome-wide cluster-based analyses and explicit introgression tests performed on the available data did not support a scenario in which *O. mellianus* is nested within *O. traillii* due to recent hybridization, as hypothesized by Jønsson et al. (2019). Instead, given the observed genome-wide signal that consistently positions *O. mellianus* in close relationship to continental *O. traillii* subspecies, it seems uncontested that they truly share recent common ancestry.

### Taxonomic Implications

Our findings suggest that the current taxonomic classification does not align with the evolutionary history of the group. *O. traillii* is paraphyletic and most of the subspecies within this complex have been reproductively isolated for a long time, certainly longer than the currently recognized species *O. mellianus* and the subspecies *O. t. traillii*. In other words, from a phylogenetic perspective, different *O. traillii* subspecies should be elevated to full species if *O. mellianus* is treated as a distinct species, which is warranted, given the lack of extant gene flow. We did not intend to revalidate or describe new species in this contribution. However, given the observed inconsistency, we use the population genetic and phylogenetic results provided by whole nuclear and mitochondrial genomes to suggest a tentative taxonomic classification in which *O. t. traillii* and *O. t. robinsoni* are elevated to species rank. While we strongly suspect that the two island subspecies of the maroon oriole form reciprocally monophyletic lineages, more data are needed to make that statement and thus, we recommend to provisionally consider them as a single species for which the name *O. t. ardens* has priority.

We note that our geographic and genetic sampling is exhaustive, but a proper assessment of species limits should draw on genetic as well as ecological, morphological, behavioral, and acoustic data (Tobias et al. 2010). Hence, we do not include a full taxonomic description in this paper, and recommend considering such information in greater detail before formally describing new species within *O. traillii*. Interestingly, despite being more homogeneous in terms of appearance, *O. traillii* subspecies do exhibit some geographic variation in plumage tones, size, bill shape, and migratory behavior (Vigors 1832; Swinhoe 1870; Stresemann 1922; Delacour 1927). An integrative taxonomic approach, which our study is one part of, is urgently needed and may lead to a significant reassessment of the conservation status of several (sub-)species (see below).

### Color genes under selection

Asian Orioles revealed marked differences in the rate and degree of phenotypic diversification and *O. mellianus* has developed its unique plumage coloration relatively recently. This observation raises the question whether color differentiation promoted by sexual selection has driven the divergence of *O. mellianus*. Plumage coloration, particularly in exposed patches such as the breast and the mantle, plays important roles in social signaling and mate choice in many birds (Hill and McGraw 2006a, 2006b). *O. traillii* males are coated with different tones of crimson-maroon depending on the subspecies and *O. mellianus* males are coated silvery white whereas females of both taxa resemble a somewhat duller version of the males (Del Hoyo et al. 2008). Therefore, plumage coloration is probably subject to female preference. One can thus speculate that color-assortative mate choice was involved in the initial separation of *O. mellianus*.

By comparing the genome-wide landscape of differentiation between *O. mellianus* and *O. t. traillii*, we identified strong candidate genes that may be important for the development of the distinct plumage of *O. mellianus*. Several of them were already previously found to be involved in avian plumage coloration (Fig. 4). However, determining how a genotype generates particular plumage phenotypes is difficult. Confounding factors such as demographic history and heterogeneous mutation rates, recombination rates, and background selection across the genome can obscure real signature selection or create false-positive signals (Ravinet et al. 2017). Furthermore, plumage-color difference in this omnivorous-species group can also be attributable to variation in dietary preference (LaFountain et al. 2013) or regulated by an interaction between multiple genes (Roulin and Ducrest 2013). A detailed understanding of the genetic basis behind the plumage differences is beyond the scope of the present study, but the identification of several candidate loci that match plumage differentiation in other avian systems is promising (Poelstra et al. 2014; Toews et al. 2016; Campagna et al. 2017). We hope that our results stimulate further attempts to understand the drivers of speciation among orioles and that they serve as a starting point for more thorough studies on the evolution of the marked color differences between *O. mellianus* and *O. traillii*.

### Implications for conservation

With the recent update of China’s endangered-species list, *O. mellianus* is now legally protected on a national level in China and Thailand as it is threatened by habitat loss and continuing declines appear to be occurring in most regions. Unfortunately, to our knowledge, no active conservation-management program is specifically targeting this species. Nonetheless, there is an urgent need to design a systematic monitoring scheme to clarify habitat and conservation requirements in order to design a suitable recovery plan (BirdLife International 2016). By outlining the genetic uniqueness and species status of this taxon, we hope to encourage authorities to intensify the protection of suitable habitat in order to preserve the remaining *O. mellianus* populations. Furthermore, we hope to promote further research focused on obtaining crucial knowledge for conservation, such as the distribution, demography, life history, and ecology of this species.

Moreover, the endemic *O. t. ardens* is currently estimated to have a declining population size of 200–500 breeding pairs (Fang 2005) and is considered endangered by the Taiwan Wildlife Conservation Act. We hope that our findings highlight the urgency for conservation prioritization since they, together with *O. t. nigellicauda*, present a deeply divergent lineage. As most conservation programs still rely on taxonomic (species) designations and our study revealed that the taxonomy currently underestimates the lineage diversity of *O. traillii* subspecies, it is urgent to re-evaluate current conservation schemes so that prioritization is not only proportional to phenotypic differences, but also considers genetic variation. Furthermore, we hope that our contribution encourages further research to study the patterns of genetic differentiation between *O. t. ardens* and *O. t. nigellicauda* since this could have important implications for the conservation of the Taiwanese maroon oriole subspecies *O. t ardens*.

## Conclusion

Understanding the evolutionary history and processes that have shaped extant populations is critical for conservation. However, it is often challenging to conduct such studies with sampling being a major hurdle (Thompson 2013). Here, we show how modern genomic technologies and NHC can be combined to reveal hidden diversity, evaluate population connectivity, and how this can ultimately guide conservation considerations. Whereas the integration of historical specimens has been long hampered by a lack of genomic resources, we demonstrate that it is now feasible and cost-effective to generate de novo reference genomes that can guide evolutionary analyses needed for both taxonomic and conservation purposes. Finally, our findings illustrate that an integrative approach is needed to identify taxonomic units for conservation, that species delineation solely based on morphology can underestimate genetic divergence, and that such studies remain vital even in relatively well-known organismal groups such as avian vertebrates.

## Supplementary information

Supplementary material

Table S1

Table S2

Table S3

## Data Availability

The raw resequenced data, the 10X chromium reads, and the 10X reference assembly have been deposited with Dryad (https://datadryad.org/stash/share/bJR2tGU20m2wZ2mWQ2z1MPik7eBkd6aXlgi3ZX341LA).
