# Supplementary material for: Utilizing museomics to trace the complex history and species boundaries in an avian-study system of conservation concern
Source: Heredity (Edinb). 2022 Jan 26;128(3):159–68. doi: 10.1038/s41437-022-00499-0 (PMC8897408; doi:10.1038/s41437-022-00499-0)
Supplement: Supplementary file 1 — Supplementary material [file 41437_2022_499_MOESM1_ESM.pdf]

Supplemental Material

Figures S1-7 and their corresponding descriptions are present in this document.

Table descriptions for tables S1-3 are present in this document.

Tables S1-3 are available in:

Table_S1.xlsx

Table_S2.xlsx

Table_S3.xlsx

Figure S1

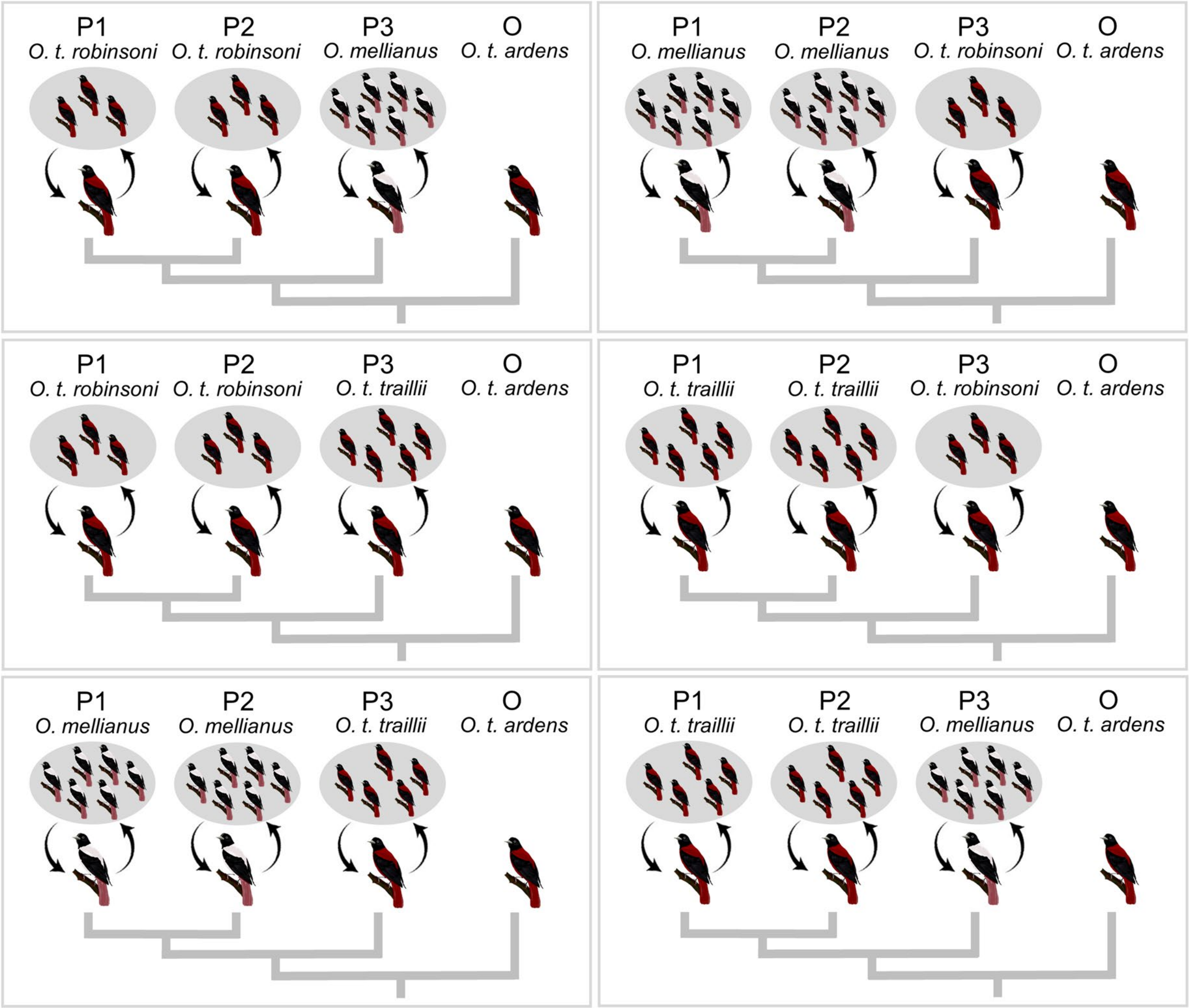

Supplementary Fig. 1: Graphic representation of how the individual-based ABBA-BABA tests were performed. The outgroup was formed by *O. t. ardens* (represented by Taiwan_B1210). When testing for excessive allele-sharing at an individual level, each leave in the tree (P1, P2, P3, O) was represented by a single individual. Individuals assigned to P1 and P2 always were conspecifics while P3 consisted of an individual representing a different taxon. In order to test for all possible comparisons, individuals were swapped so that all individuals within each taxon were compared against each other and against each of the samples belonging to the different taxon.

Figure S2:

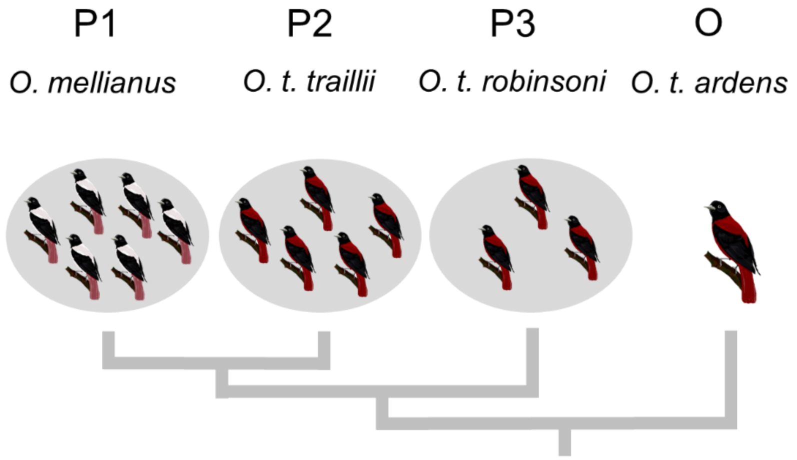

Supplementary Fig. 2: Graphic representation of how the population-level ABBA-BABA test was performed. The outgroup was formed by *O. t. ardens* (represented by Taiwan_B1210). As opposed to the individual-based tests, each leave in the tree (P1, P2, P3, O) is represented by multiple samples simultaneously. This design aims to test whether the entire population of *O. t. traillii* collectively shares an excessive amount of derived alleles with *O. t. robinsoni* compared to *O. mellianus*.

Figure S3:

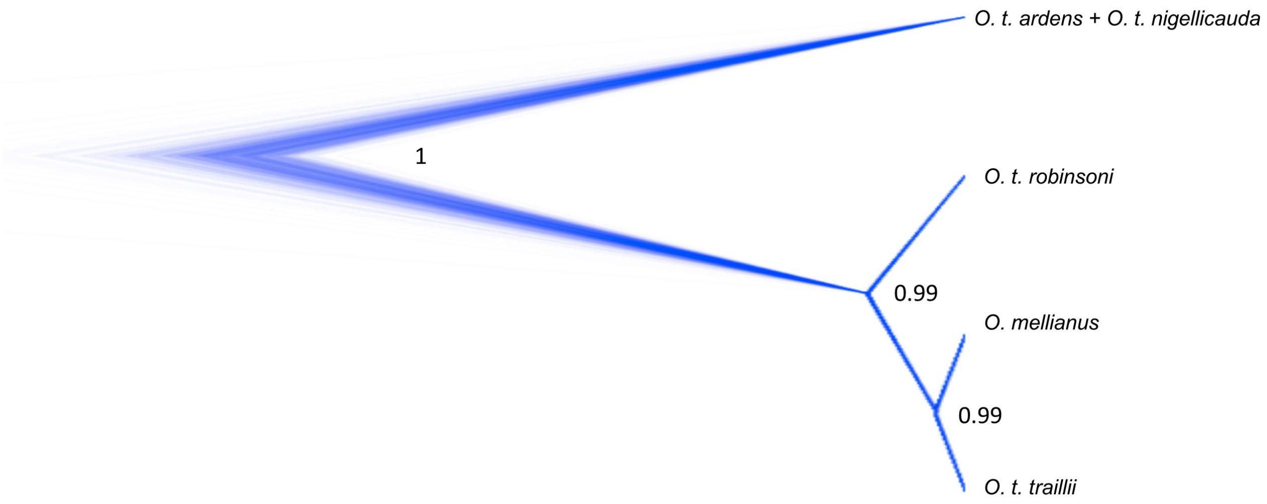

Supplementary Fig. 3: DensiTree plot showing 1,500 trees sampled from the SNAPP posterior species-tree distribution. The SNAPP analysis was conducted on 84,236 nuclear SNPs.

Figure S4:

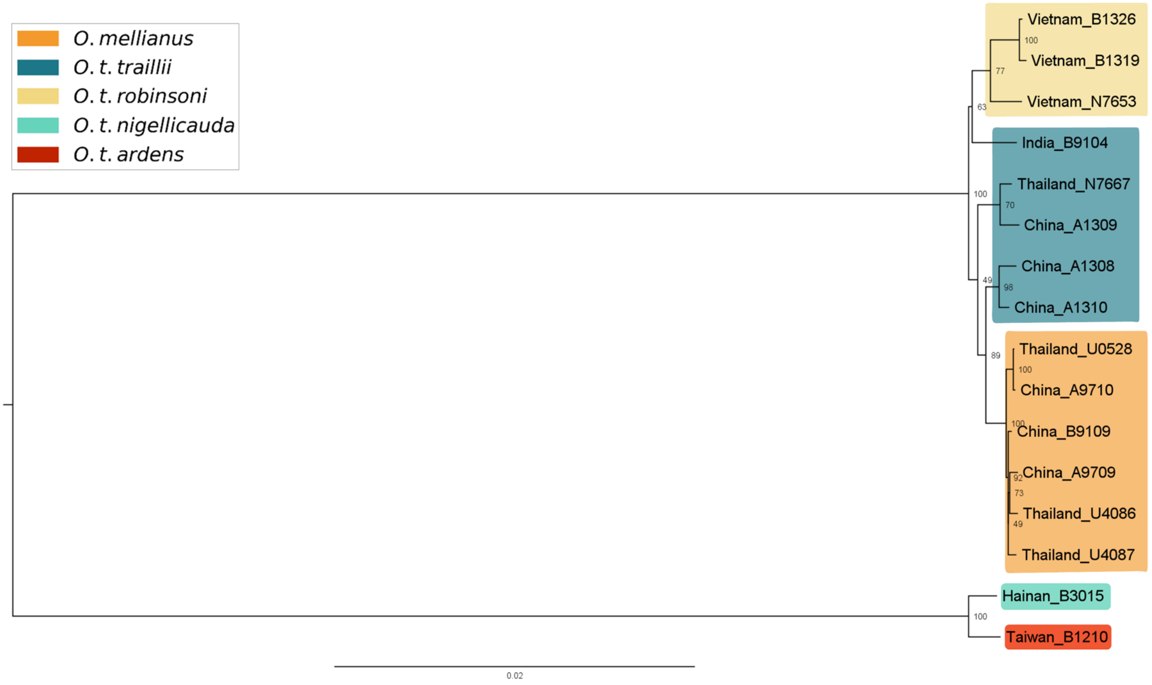

Supplementary Fig. 4: Best-scoring maximum-likelihood tree obtained from full mitogenome sequences (excluding the control region). The scale bar (0.02) is measured in absolute divergence-time units.

Figure S5:

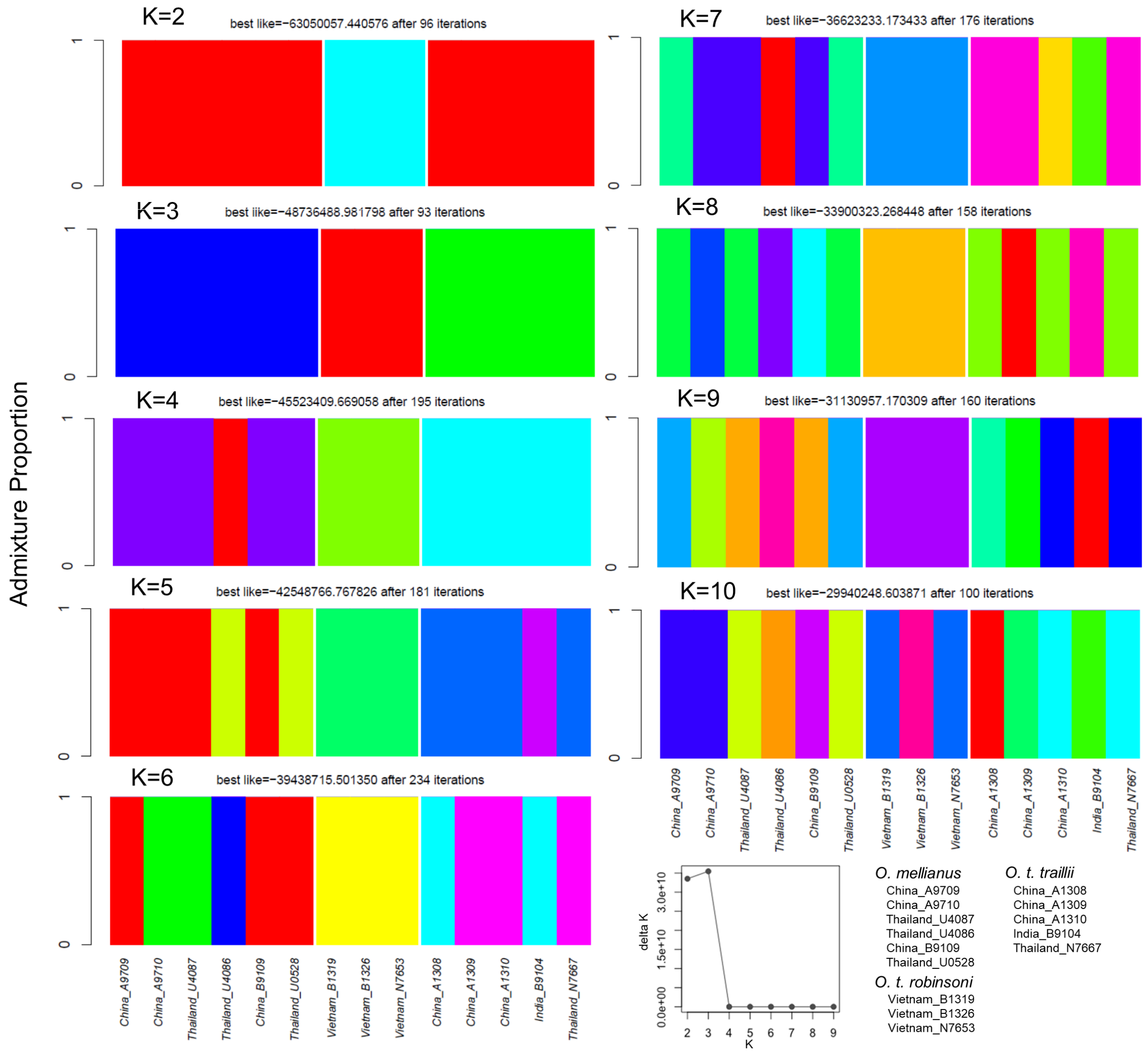

Supplementary Fig. 5: NGSadmix results obtained based on the continental taxa (*O. mellianus*, *O. t. traillii* and *O. t. robinsoni*). The analyses were based on nuclear genomic SNP data. The admixture panels show the best scoring cluster configurations out of 15 runs under K ranging from 2 to 10. The Y-axis represents the admixture proportion and the X-axis indicates the sample names. Thick white lines draw the taxon boundaries according to the current taxonomic classification. Plots to the lower right shows how DeltaK changes as the K-value increases. The optimal number of discrete groups returned by DeltaK is 3. The legend in the lower right shows the taxon-correspondence of each sample.

Figure S6:

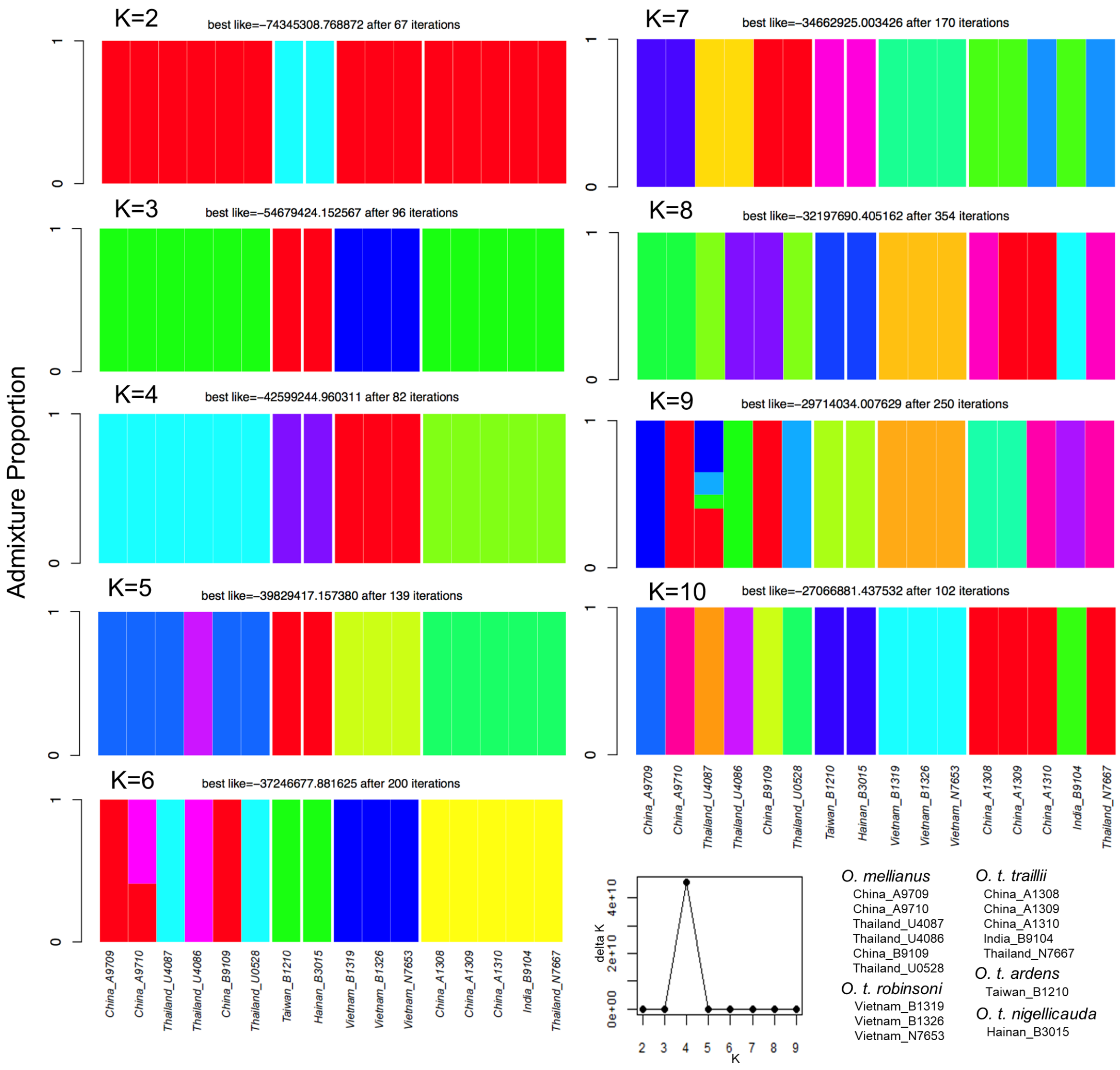

Supplementary Fig. 6: NGSadmix results obtained when including all the samples. The analyses were based on nuclear genomic SNP data. The admixture panels show the best-scoring cluster configurations out of 15 runs under K ranging from 2 to 10. The Y-axis represents the admixture proportion and the X-axis indicates the sample names. Thick white lines draw the taxon boundaries according to the current taxonomic classification. The optimal number of discrete groups returned by DeltaK is 4. The legend in the lower right shows the taxon-correspondence of each sample.

Figure S7:

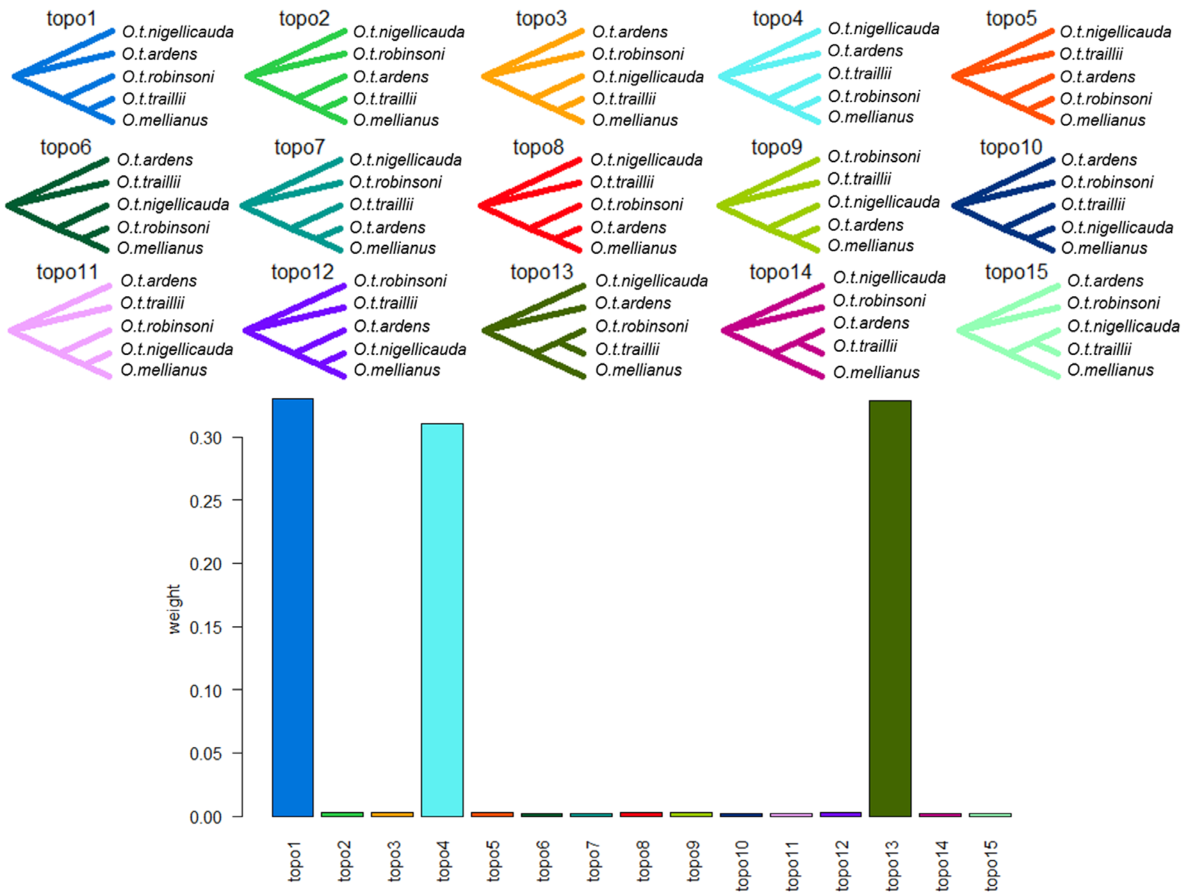

Supplementary Fig. 7: Gene-tree frequency analysis using Twisst. The phylogenies on the top show the topologies that have been weighted. The bottom bar plot shows the relative frequency of the different topologies. Notice that topologies 1, 4, and 13 reflect the three possible in-group topologies (involving continental taxa) and are almost equally frequent (supported by ~30% of the gene-trees).

Tables S1-3:

Table S1: Species identification and meta-data for the individuals included in the study. Sample ID assigned in the present study; Voucher/Museum Code; Sampling Location; Year of Collection; Number of Raw Reads; Number of Cleaned reads; Number of Mapped Reads; % C to T, observed frequency of C to T transitions at the first position of the 5' end of reads; % G to A, observed frequency of G to A transitions at the first position of the 3' end of reads.

Table S2: P-distances calculated based on a concatenated dataset of nuclear genomic regions. Samples are color-coded according to the coloration assigned throughout the paper.

Table S3: Population and individual-level D-statistics obtained by the ABBA-BABA tests. |Z|>3 or p-value<0.005 is considered significant. Samples are color coded according to the coloration assigned throughout the paper.
